# Cardiovascular magnetic resonance characteristics and clinical outcomes of patients with ST-elevation myocardial infarction and no standard modifiable risk factors–A DANAMI-3 substudy

**DOI:** 10.3389/fcvm.2022.945815

**Published:** 2022-08-03

**Authors:** Jawad Mazhar, Kathrine Ekström, Rebecca Kozor, Stuart M. Grieve, Lars Nepper-Christensen, Kiril A. Ahtarovski, Henning Kelbæk, Dan E. Høfsten, Lars Køber, Niels Vejlstrup, Stephen T. Vernon, Thomas Engstrøm, Jacob Lønborg, Gemma A. Figtree

**Affiliations:** ^1^Kolling Research Institute, University of Sydney, Sydney, NSW, Australia; ^2^Department of Cardiology, The Heart Centre, Rigshospitalet, Copenhagen University Hospital, Copenhagen, Denmark; ^3^Imaging and Phenotyping Laboratory, Faculty of Medicine and Health, Charles Perkins Centre, University of Sydney, Sydney, NSW, Australia; ^4^Department of Radiology, Royal Prince Alfred Hospital, Camperdown, NSW, Australia; ^5^Department of Cardiology, Zealand University Hospital, Roskilde, Denmark; ^6^Department of Cardiology, Lund University Hospital, Lund, Sweden

**Keywords:** coronary artery disease, ST elevation myocardial infarction, cardiovascular risk factors, atherosclerosis, cardiovascular magnetic resonance

## Abstract

**Introduction:**

A higher 30-day mortality has been observed in patients with first-presentation ST elevation myocardial infarction (STEMI) who have no standard modifiable cardiovascular risk factors (SMuRFs), i. e., diabetes, hypertension, hyperlipidemia, and current smoker. In this study, we evaluate the clinical outcomes and CMR imaging characteristics of patients with and without SMuRFs who presented with first-presentation STEMI.

**Methods:**

Patients from the Third DANish Study of Acute Treatment of Patients With ST-Segment Elevation Myocardial Infarction (DANAMI-3) with first-presentation STEMI were classified into those with no SMuRFs vs. those with at least one SMuRF.

**Results:**

We identified 2,046 patients; 283 (14%) SMuRFless and 1,763 (86%) had >0 SMuRF. SMuRFless patients were older (66 vs. 61 years, *p* < 0.001) with more males (84 vs. 74%, *p* < 0.001), more likely to have left anterior descending artery (LAD) as the culprit artery (50 vs. 42%, *p* = 0.009), and poor pre-PCI (percutaneous coronary intervention) TIMI (thrombolysis in myocardial infarction) flow ≤1 (78 vs. 64%; *p* < 0.001). There was no difference in all-cause mortality, non-fatal reinfarction, or hospitalization for heart failure at 30 days or at long-term follow-up. CMR imaging was performed on 726 patients. SMuRFless patients had larger acute infarct size (17 vs. 13%, *p* = 0.04) and a smaller myocardial salvage index (42 vs. 50%, *p* = 0.02). These differences were attenuated when the higher LAD predominance and/or TIMI 0-1 flow were included in the model.

**Conclusion:**

Despite no difference in 30-day mortality, SMuRFless patients had a larger infarct size and a smaller myocardial salvage index following first-presentation STEMI. This association was mediated by a larger proportion of LAD culprits and poor TIMI flow pre-PCI.

**Clinical trial registration:**

clinicaltrials.gov, unique identifier: NCT01435408 (DANAMI 3-iPOST and DANAMI 3-DEFER) and NCT01960933 (DANAMI 3-PRIMULTI).

## Introduction

Despite the common perception that coronary artery disease (CAD) is well understood and managed, it remains the leading cause of mortality in adults worldwide (1). Major advances have been made in the identification and treatment of standard modifiable risk factors (SMuRFs) for CAD—particularly hypercholesterolemia, hypertension, diabetes mellitus, and smoking (2, 3). However, at an individual level, it is not uncommon for a patient to present with extensive atherosclerosis and acute coronary syndrome that is not clearly explained by such risk factors. We have previously reported an increase in the prevalence of myocardial infarction patients presenting with no standard modifiable cardiovascular risk factors in Australia from 11 to 27% over a 10-year period (4), confirmed in a large, nation-wide cohort (5).

A number of large observational studies have shown, somewhat unexpectedly, that SMuRFless STEMI patients have higher in-hospital and 30-day mortality rates after myocardial infarction compared to patients with at least one of the major risk factors (4–9). A recent analysis of 62,048 patients with first presentation ST-segment elevation myocardial infarction (STEMI) in the SWEDEHEART (Swedish Healthcare Registry on Heart Disease) registry showed that SMuRFless patients had a ~1.5 times higher 30-day mortality rate compared to patients with >0 SMuRFs (9). Shuffles women had the highest 30-day mortality (18%), with the heightened susceptibility factors presumably driving atherosclerosis compounding their already recognized poor outcomes post-STEMI compared with men (9). The reasons for these higher mortality rates in SMuRFless patients following myocardial infarction are not well described, but current data suggests they may be driven by arrhythmia rather than reinfarction or heart failure (9). Differences in myocardial infarct size and microvascular obstruction (MVO) may also contribute to the higher 30-day mortality rates, but this remains to be investigated.

Cardiac magnetic resonance (CMR) imaging allows direct quantitation of the area at risk (AAR), myocardial infarct size, and MVO. Both myocardial infarct size and MVO are associated with poor outcome (10–12). CMR has also been employed to evaluate the impact of medical therapies on left ventricular remodeling post-myocardial infarction (13–16). In this study, we evaluate the clinical outcome and CMR measures of infarct characteristics and LV remodeling in patients with and without SMuRFs who presented with first-presentation STEMI.

## Methods

### Study population

The Third DANish Study of Optimal Acute Treatment of Patients With ST-Segment Elevation Myocardial Infarction (DANAMI-3) trial programme (NCT 01960933, NCT01435408) consisted of three randomized controlled STEMI trials performed at four large primary percutaneous coronary intervention (PCI) centers in Denmark between March 2011 and February 2014 (17–20). The studies examined the effect of ischemic postconditioning (iPOST), deferred stenting (DEFER), and fractional flow reserve (FFR)-guided complete revascularization (PRIMULTI) in patients following STEMI. Patients were primarily randomized to either iPOST or DEFER. A secondary randomization was subsequently performed, randomizing patients with multivessel disease (MVD) in the PRIMULTI trial. For the current study, patients from all the DANAMI-3 trials with first-presentation STEMI were classified into patients with no SMuRFs vs. those with at least one SMuRF, to assess for any significant differences in clinical outcomes. SMuRFless patients were defined as patients with no standard modifiable cardiovascular risk factors—diabetes mellitus, hypertension, hyperlipidaemia, and current smoker. A patient was classified as having diabetes, hypertension, or hyperlipidaemia if the patient reported a history of such, was on the treatment of these conditions at the time of presentation, or LDL at the time of admission was ≥3.5 mmol/L. A patient was defined as a current smoker if they reported being smoking regularly prior to admission. The clinical outcomes of the two groups were compared as per the primary and secondary endpoints defined in the DANAMI-3 trial (17–20). Baseline clinical characteristics, procedural data, medications at discharge as well as clinical outcomes were obtained.

### The CMR sub-study

**Figure 1:**
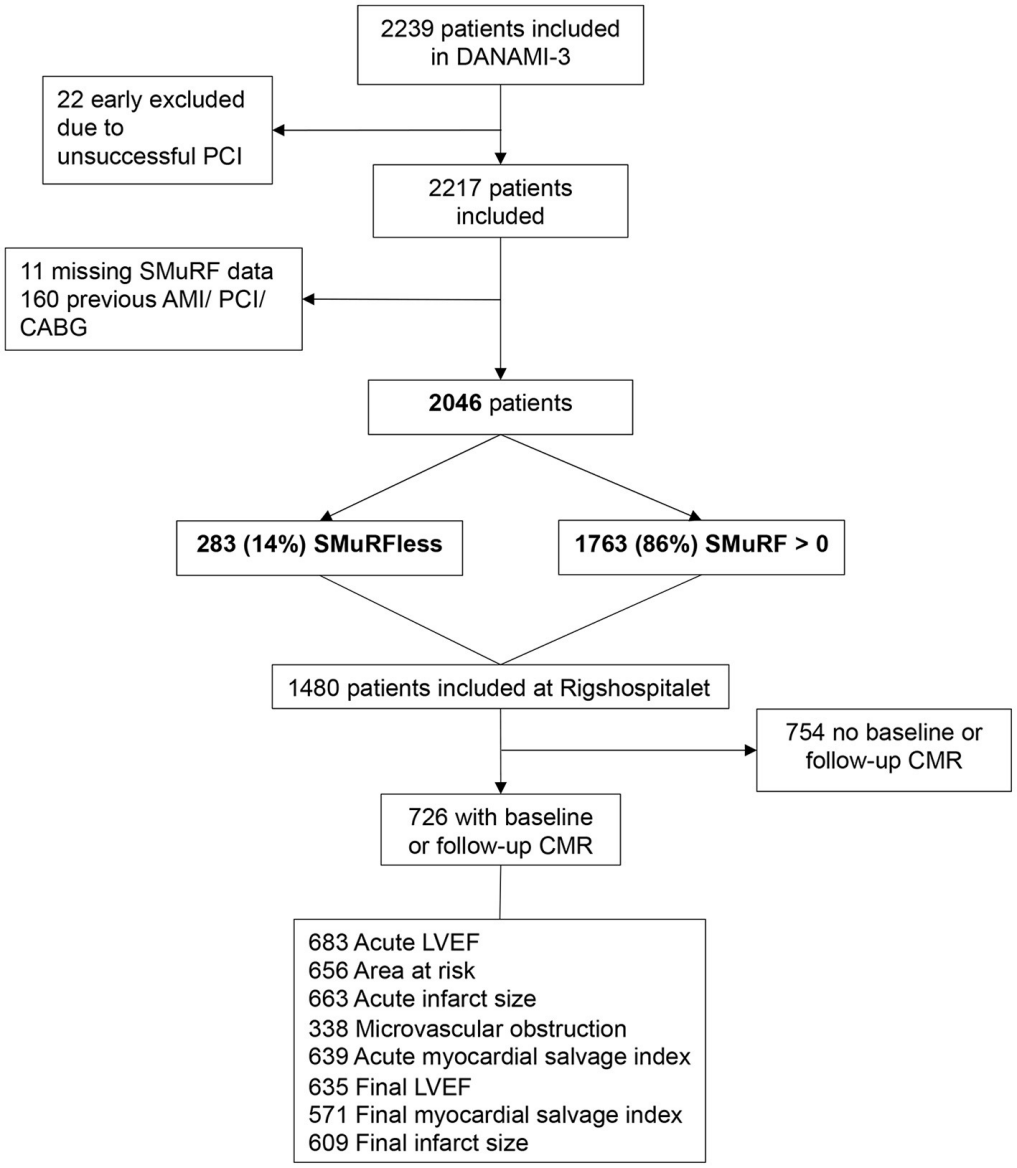
Flowchart.

A subgroup of DANAMI-3 patients recruited at one center (Righospitalet, Copenhagen University Hospital) had CMR performed according to a standard protocol to evaluate the effects of randomizations on a surrogate marker. This CMR-subgroup provided an optimal opportunity to examine any potential differences in CMR variables such as AAR, infarct size, MVO, and LV remodeling between individuals with no SMuRFs vs. those with at least one SMuRF. Patients were included in the sub-study if they had baseline and/or follow-up CMR performed (Figure 1).

#### Acquisition and analysis of CMR images

The CMR protocol has been described in detail previously (21). Briefly, CMR was performed during index admission following primary PCI using a 1.5 Tesla scanner (Avanto; Siemens, Erlangen, Germany). CMR was also repeated 3 months after the index admission. Images were analyzed by an independent observer, blinded to all clinical data, using dedicated software (CVI42, Circle Cardiovascular Imaging Inc, Calgary, Alberta, Canda), and validated by a second experienced independent observer. Left ventricular function and mass were calculated on short-axis steady-state free precession (SSFP) cine images on index and follow-up CMRs. Infarct size was assessed on late gadolinium enhancement images, which were obtained 10 min after intravenous injection of 0.1 mmol/kg body weight of gadolinium-based contrast (Gadovist; Bayer Schering, Berlin, Germany) on 8 mm sliced short-axis images with full-LV coverage and no gap (14). Myocardial infarction was defined as hyperenhanced myocardium with a signal intensity of >5 SDs of the mean intensity of normal reference myocardium. Infarct size was calculated as a percentage of left ventricular mass and reported on both index and follow-up CMRs. AAR was identified as oedema on the index scan using a T2-weighted short tau inversion-recovery sequence (STIR) (13, 15). Oedema was defined as an area with a signal intensity of >2 SDs above the signal intensity in remote normal myocardium. Microvascular obstruction (MVO) was assessed on the LGE images in the index scans as hypointense areas within the acute infarct region. Myocardial salvage index (MSI) was calculated as the difference between area at risk and infarct size, divided by area at risk (22).

### Statistical analysis

The DANAMI-3 patients with first-presentation STEMI were identified and classified into patients with no SMuRF (SMuRFless) and those with at least one SMuRF. Continuous variables were compared using Student's *t*-test (if normal distribution) or Mann–Whitney *U* test (if not normal distribution). Mean ± SD or median (interquartile range, IQR) or percentages are reported. Changes in baseline to follow-up CMR measures were assessed using the paired *t*-test. Categorical variables were compared using Pearson's chi-squared or Fisher's exact test as appropriate. Multivariable linear regression analyses were performed using SMuRFless patients as a predictor for final infarct size or final MSI adjusting for pre-specified clinically relevant variables: male sex, age, ECG–to-wire time, former smoking, thrombolysis in myocardial infarction (TIMI) flow 0–1 pre-PCI and culprit left anterior descending artery (LAD). A Cox proportional hazards regression model was used to assess the individual risk of a clinical outcome in SMuRFless patients. Finally, an additional logistic regression model was performed to determine the independent predictors of TIMI 0-1 flow, adjusting for pre-specified clinically relevant variables, namely, male sex, age, symptom to wire time, multivessel disease, culprit LAD, heart rate, systolic blood pressure, (23) and SMuRFless status. A result was considered significant when it was below a two-sided *p*-value of 0.05. The statistical analyses were performed with SPSS version 25.

## Results

### Study population

A total of 2,217 patients were included in the DANAMI-3 trial program. After excluding 160 patients with previous acute myocardial infarction, PCI, and coronary artery bypass grafting (CABG), and 11 patients with missing SMuRF data, the total study population was comprised of 2,046 patients. Of these, 283 (14%) were SMuRFless and 1,763 (86%) had >0 SMuRFs. A total of 726 (13%) patients underwent baseline and/or follow-up CMR imaging: 688 (95%) had CMR at baseline and 639 (88%) had CMR at follow up. A total of 87 patients had only baseline CMR and 38 had only follow-up CMR performed. Of the 726 patients in the CMR sub-group, 61 (8%) were SMuRFless, and 665 (92%) had >0 SMuRFs (Figure 1).

In the CMR subgroup, the proportion of SMuRF-less and SMuRF patients in the three randomized trials was similar (Appendix Table A1). In the total DANAMI-3 population, the proportion of SMuRFless and SMuRF patients was similar in PRIMULTI and iPOST but not similar in DEFER (Appendix Table A1). Additional analyses were performed to ensure, that there was no effect of randomizations on the results. There was no interaction between SMuRFs and either of the treatment arms in DANAMI-3 with regards to acute infarct size (DEFER *p* = 0.45, iPOST *p* = 0.70, and PRIMULTI *p* = 0.89).

### Baseline clinical characteristics

The baseline clinical characteristics for the total population and the CMR subgroup are presented in Table 1. In the total study population, SMuRFless patients were older with more males, a larger proportion were former smokers, but fewer had a family history of ischemic heart disease. In the CMR sub-group, the SMuRFless patients were older, but the proportion of males was similar and likewise, a larger proportion were former smokers.

**Table 1:** Baseline characteristics between SMuRFless and patients with more than one SMuRF, total DANAMI-3 and CMR sub-population.

	**Total population**			**CMR sub-population**		
**Parameter**	**SMuRFless** ***n* = 283 (14%)**	**SMuRF > 0** ***n* = 1,763 (86%)**	***p*-value**	**SmuRFless** ***n* = 61 (8%)**	**SMuRF > 0** ***n* = 665 (92%)**	***p*-value**
Age, years	66 ± 12	61 ± 12	<0.001	63 ± 11	58 ± 11	0.003
Male, %	238 (84)	1,322 (75)	<0.001	51 (84)	523 (79)	0.42
SMuRFs						
Hypertension, %	0 (0)	758 (43)		0 (0)	245 (37)	
Diabetes, %	0 (0)	172 (10)		0 (0)	59 (9)	
Hyperlipidemia, %	0 (0)	1,019 (73)		0 (0)	487 (74)	
Current smoker	0 (0)	1,043 (59)		0 (0)	395 (59)	
Former smoker	148 (52)	420 (24)	<0.001	34 (56)	115 (23)	<0.001
Pre-existing chronic heart failure, %	69 (24)	312 (18)	0.007	15 (25)	105 (16)	0.08
Pre-existing chronic kidney disease, %	1 (1)	25 (2)	0.33	0 (0)	4 (1)	0.52
Pre-existing stroke, %	3 (2)	59 (4)	0.24	2 (3)	21 (3)	0.96
BMI, kg/m^2^	27 ± 4	27 ± 4	0.95	28 ± 4	27 ± 4	0.11
Family history of IHD, %	93 (35)	785 (46)	0.001	26 (43)	328 (50)	0.35
Time from first ecg to wire, minutes	87 (73–118)	87 (71–114)	0.64	91 (76–115)	85 (69–114)	0.31
Admission eGFR < 60 mL/min/1,73 m^2^, %	16 (12)	140 (11)	0.75	1 (2)	29 (5)	0.30
Admission Hgb < 6.0 mmol/L, %	4 (1)	11 (1)	0.15	1 (2)	2 (0.3)	0.12

### Procedural characteristics

Procedural characteristics for both populations are presented in Table 2. In the total study population and the CMR sub-group, the SMuRFless and SMuRF patients were similar in terms of the number of arteries treated, number of implanted stents, stent diameter, total stent length, and the use of periprocedural medications such as heparin, glycoprotein IIb/IIIa, and bivalirudin. In the total study population, the number of patients with multivessel disease (MVD) was less in the SMuRFless group, however, in the CMR sub-group the number of patients with MVD was similar. The proportion of patients with TIMI flow 0–1 before PCI was higher in the SMuRFless group in both the total study population (78 vs. 64%, *p* = 0.001) and the CMR subgroup (75 vs. 61%, *p* = 0.02). In the total study population, the left anterior descending artery (LAD) was the culprit artery in a higher proportion of SMuRFless patients (50 vs. 42%, *p* = 0.009). In the CMR sub-group, the proportion was not significantly different (44 vs. 41%, *p* = 0.60).

**Table 2:** Procedural data and medication given at discharge, total DANAMI-3 and CMR sub-population.

	**Total population**			**CMR sub-population**		
	**SMuRFless *n* = 283 (14%)**	**SMuRF > 0** ***n* = 1,763 (86%)**	***p*-value**	**SmuRFless** ***n* = 61 (8%)**	**SMuRF > 0** ***n* = 665 (92%)**	***p*-value**
No. of main arteries treated per patient, mean_*a*_	1 (1–1)	1 (1–1)	0.89	1 (1–1)	1 (1–1)	0.85
No. of implanted stents, *n* (IQR)	1 (1–2)	1 (1–2)	0.80	1 (1–2)	1 (1–2)	0.81
Stent diameter, mean, mm	3.4 ± 0.5	3.5 ± 0.5	0.52	3.4 ± 0.7	3.5 ± 0.6	0.21
Total stent length, mm (IQR)	18 (15–33)	23 (15–33)	0.80	21 (18–36)	23 (18–33)	0.87
Stent type			0.16			0.70
None	1 (0)	21 (1)		0 (0)	8 (1)	
POBA	16 (6)	79 (5)		4 (7)	50 (5)	
Bare-metal	6 (2)	37 (2)		1 (2)	12 (2)	
Drug-eluting	258 (92)	1,607 (92)		55 (92)	607 (92)	
Pretreatment with heparin	268 (96)	1,645 (96)	0.94	58 (97)	606 (95)	0.56
Use of glycoprotein IIb/IIIa inhibitor	49 (18)	318 (19)	0.68	15 (25)	139 (22)	0.57
Use of bivalirudin	220 (79)	1,308 (76)	0.38	43 (72)	463 (73)	0.88
Thrombus aspiration	158 (56)	1,016 (58)	0.57	39 (64)	392 (59)	0.45
Killip class III-IV heart failure at any time	6 (2)	25 (1)	0.37	0 (0)	3 (1)	0.60
Multivessel disease	90 (32)	709 (40)	0.006	21 (34)	280 (42)	0.23
Location of culprit lesion						
LM	0 (0)	3 (0)	0.49	0 (0)	1 (0)	0.76
LAD	142 (50)	736 (42)	0.009	27 (44)	271 (41)	0.60
RCA	104 (37)	759 (43)	0.04	22 (36)	293 (44)	0.22
LCx	37 (13)	261 (15)	0.44	12 (20)	99 (15)	0.32
TIMI flow grade 0/1 before PCI^b^	222 (78)	1,130 (64)	<0.001	46 (75)	402 (61)	0.02
TIMI flow grade 2/3 after PCI^b^	282 (99)	1,752 (99)	0.58	61 (100)	661 (99)	0.54
Radial access	8 (3)	113 (7)	0.02	3 (5)	44 (7)	0.58

Results expressed as mean±SD, interquartile range (IQR), or percentage. LCx, left circumflex artery; LAD, left anterior descending artery; LM, left main artery; POBA, plain old balloon angioplasty; RCA, right coronary artery; TIMI, thrombolysis in myocardial infarction.

a Includes diagonal, obtuse marginal, posterolateral coronary artery, and posterior descending coronary artery.

b Grades range from 0 to 3, with 3 indicating higher flow.

### Discharge medications

Appendix Table A2 summarizes discharge medications for both groups. In the total population, a significantly higher proportion of SMuRFless patients were discharged with clopidogrel or ticagrelor treatment vs. a higher proportion of patients with SMuRFs treated with prasugrel. No substantial group-level differences in the CMR subgroup were demonstrated.

### CMR endpoints

Of the 726 patients in the CMR sub-group, 688 (95%) patients had a baseline CMR performed at a median of 0 days [IQR: 0–1] after primary PCI, and 639 (88%) patients had a follow-up CMR performed after a median of 90 days [IQR: 88–96]).

#### Infarct size, microvascular obstruction, and myocardial salvage index

**Figure 2:**
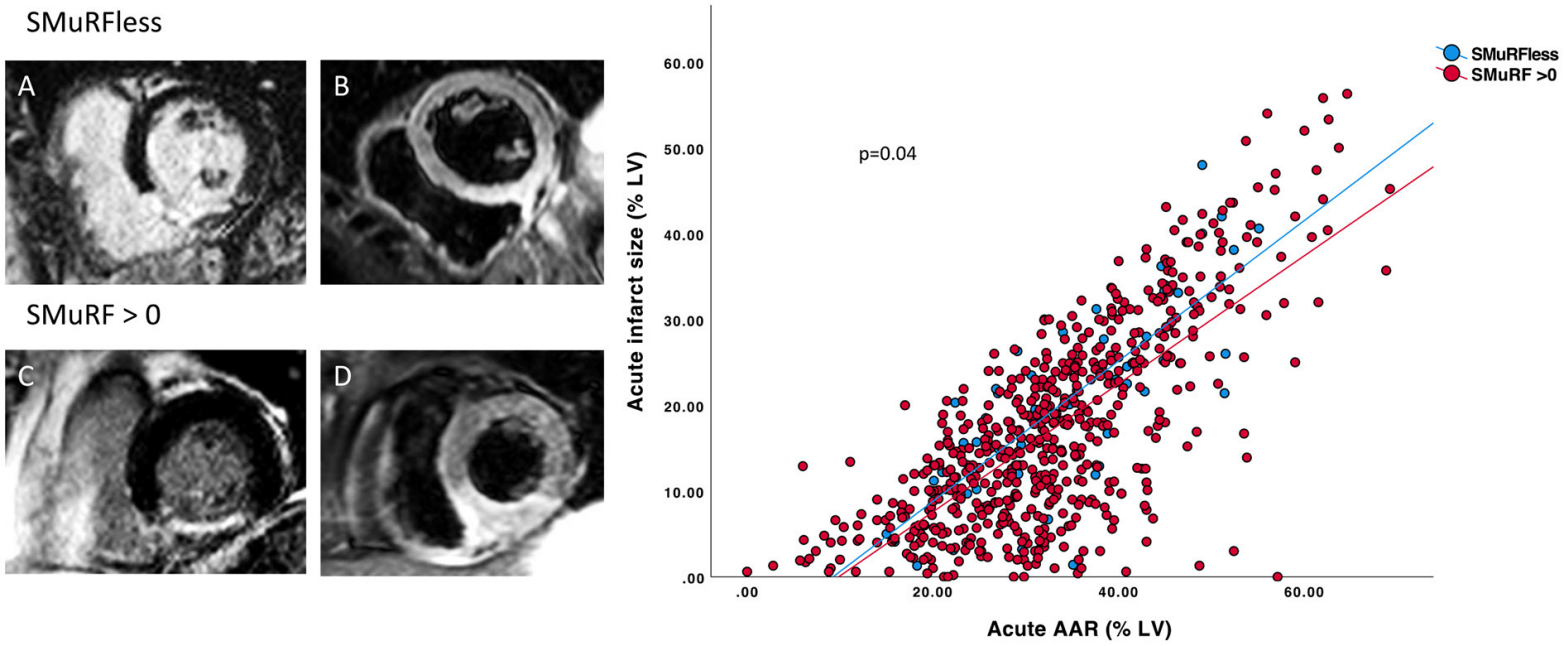
Relationship between infarct size and area at risk between SMuRFless and SMuRF >0 individuals. Representative CMR images of mid-ventricular LGE and T2-weighed STIR slices at baseline. SMuRFless patient: **(A)** LGE contrast enhanced slice showing area with hyperintense signal in the inferior-septal region indicating infarction. **(B)** T2-weighed STIR slice showing hyperintense signal in the same region indicating the AAR (oedema) and thus acute infarction. Same images are shown for a patient with SMuRF >0: **(C)** LGE image of the infarction with corresponding **(D)** T2-weighed STIR image of the AAR. Scatterplot (right panel) of the acute infarct size and AAR in SMuRFless vs. SMuRF patients showing a larger infarct size in SMuRFless patients after adjustment for AAR.

CMR results are presented in Table 3. At baseline, SMuRFless patients had significantly larger acute and final infarct sizes with a similar-sized AAR compared to their counterparts. After adjusting for the AAR, there remained an association between larger acute infarct size and SMuRFless status (B 2.3% LV mass, 95% CI (0.1–4.4%), *p* = 0.04) compared to patients with >0 SMuRFs. The increased infarct size to AAR is presented in Figure 2. This is reflected in the lower acute MSI in SMuRFless patients. MVO was similar to those with or without SMuRFs.

**Table 3:** CMR variables at baseline and follow-up.

**Parameter**	**SmuRFless *n* = 61 (8%)**	**SMuRF > 0** ***n* = 665 (92%)**	***p*-value**
**Baseline**			
LV EDV (ml)	172 ± 39	165 ± 38	0.20
LV EDV index (ml/m^2^)	86 ± 18	83 ± 16	0.13
LV ESV (ml)	88 ± 34	83 ± 29	0.23
LV ESV index (ml/m^2^)	44 ± 17	41 ± 14	0.22
LVEF (%)	50 ± 11	51 ± 10	0.74
Acute infarct size (% LV)^*^	17 ± 2	13 ± 2	0.04
AAR (% LV)	35 ± 10	33 ± 11	0.24
MVO (% LV)^*^	3 ± 3	3 ± 2	0.78
Acute MSI (%)	42 ± 22	50 ± 25	0.02
**Follow-up**			
LV EDV (ml)	186 ± 44	170 ± 40	0.006
LV EDV index (ml/m^2^)	93 ± 19	86 ± 18	0.006
LV ESV (ml)	83 ± 34	74 ± 30	0.03
LV ESV index (ml/m^2^)	41 ± 16	37 ± 15	0.06
LVEF (%)	57 ± 10	58 ± 9	0.42
Final infarct size (% LV)^*^	12 ± 2	8 ± 3	0.03
Final MSI (%)	60 ± 22	64 ± 24	0.23
**Δ Baseline to follow-up**			
LV EDV (ml)	13 ± 26 ^**^	5 ± 27 ^**^	0.06
LV EDV index (ml/m^2^)	6 ± 13 ^**^	3 ± 13 ^**^	0.10
LV ESV (ml)	−7 ± 21 ^**^	−9 ± 22 ^**^	0.58
LV ESV index (ml/m^2^)	−4 ± 11 ^**^	−4 ± 11 ^**^	0.69
LVEF (%)	7 ± 10^**^	7 ± 8 ^**^	0.50
Infarct size (% LV)	−1.6 ± 1.7 ^**^	−1.5 ± 1.8 ^**^	0.30
LV EDV % change from baseline – follow-up CMR	8 ± 17	4 ± 17	0.14
LV ESV % change from baseline – follow-up CMR	−5 ± 25	−8 ± 25	0.31
Patients with ≥ 12% increase in LV EDV and LV ESV from baseline to follow-up, *n* (%)	6 (11)	72 (13)	0.68

Data are expressed as mean±SD.

* Logarithm transferred.

** p-Value within groups < 0.001, paired t-test.

AAR, area at risk; EDV, end-diastolic volume; ESV, end-systolic volume; LV, left ventricular; LVEF, left ventricular ejection fraction; MVO, microvascular obstruction.

In a multivariable regression model, the association of SMuRFless status with larger acute infarct size remained significant independent of age, sex, former smoking, and ECG-to-wire time. However, this association was no longer observed if culprit LAD and/or TIMI 0-1 flow pre-PCI were included in the model (Table 4). As TIMI 0-1 flow pre-PCI was a strong predictor of infarct size, a logistic regression analysis was performed to determine the predictors of TIMI 0-1 flow pre-PCI (Table 5). This model included clinically relevant variables, namely, male sex, age, symptom-to-wire time, multivessel disease, culprit LAD, heart rate, systolic blood pressure, and being SMuRFless. The predictors of TIMI 0-1 flow pre-PCI in this model were SMuRFless status (OR 1.6, CI (1.03–2.4), *p* = 0.03), male sex (OR 1.4, CI (1.1–1.9), *p* = 0.006), culprit LAD (OR 0.7, CI (0.6–0.9), *p* = 0.005), and symptom-to-wire time (OR 1.0, CI 1.0–1.0, *p* = 0.001).

**Table 4:** Multivariable linear regression analysis for predictors of acute infarct size and MSI.

	**Acute infarct size**				**Myocardial salvage index**			
	**Univariable**		**Multivariable**		**Univariable**		**Multivariable**	
	**B**	***P*-value**	**B**	***P*-value**	**B**	***P*-value**	**B**	***P*-value**
**Model 1**								
Male sex	3.2	0.004	3.9	<0.001	−4.4	0.07	−5.3	0.04
Age, years	0.1	0.01	0.1	0.007	−0.2	0.05	−0.1	0.12
Former smoker	−0.1	0.92	−1.5	0.18	−0.4	0.87	1.7	0.48
ECG-to-wire-time, min	0.02	0.08	0.02	0.08	−0.06	0.01	−0.06	0.006
SMuRFless	3.5	0.03	3.3	0.04	−7.8	0.02	−7.7	0.03
**Model 2–TIMI flow**								
Male sex			3.7	<0.001			−4.8	0.04
Age, years			0.1	0.06			−0.1	0.16
Former smoker			−1.5	0.14			1.9	0.38
ECG-to-wire-time, min			0.02	0.02			−0.06	0.002
SMuRFless			2.2	0.15			−5.4	0.10
TIMI 0-1 pre-PCI	9.1	<0.001	8.8	<0.001	−20.7	<0.001	20.6	<0.001
**Model 3–culprit LAD**								
Male sex			3.7	0.001			−5.2	0.04
Age, years			0.1	0.03			−0.1	0.43
Former smoker			−1.0	0.35			1.5	0.52
ECG-to-wire-time, min			0.01	0.20			−0.05	0.02
SMuRFless			3.1	0.05			−7.6	0.03
Culprit LAD	5.1	<0.001	5.2	<0.001	−2.2	0.26	−2.0	0.32
**Model 4–full model**								
Male sex			3.5	<0.001			−4.7	0.04
Age, years			0.1	0.04			−0.1	0.22
Former smoker			−1.0	0.32			1.6	0.46
ECG-to-wire-time, min			0.02	0.08			−0.06	0.003
SMuRFless			1.8	0.21			5.1	0.12
TIMI 0-1 pre-PCI			9.5	<0.001			21.1	<0.001
culprit LAD			6.2	<0.001			−4.5	0.02

**Table 5:** Uni and multivariable logistic regression analysis for predictors of TIMI 0-1 flow pre-PCI.

	**Univariable**		**Multivariable**	
	**Odds Ratio** **(95% CI)**	***P*-value**	**Odds Ratio** **(95% CI)**	***P*-value**
Male sex	1.3 (1.1–1.7)	0.005	1.4 (1.1–1.9)	0.006
Age, years	1.0 (0.99–1.01)	0.86	0.99 (0.99–1.00)	0.81
Symptom-wire time, min	1.001 (1.001–1.002)	0.003	1.0 (1.0–1.0)	0.001
Multivessel disease	1.0 (0.8–1.3)	0.7	0.9 (0.7–1.2)	0.60
Culprit LAD	0.8 (0.7–0.95)	0.01	0.7 (0.6–0.9)	0.005
Heart rate, bpm	0.99 (0.98–0.99)	0.008	0.99 (0.98–1.0)	0.08
Systolic blood pressure	1.0 (1.0–1.001)	0.83	1.0 (1.0–1.0)	0.46
SMuRFless	2.0 (1.5–2.8)	<0.001	1.6 (1.03–2.4)	0.03

#### Left ventricle function and remodeling

At baseline CMR, there was no difference in indexed left ventricle end diastolic volume (LVEDV), indexed left ventricular end systolic volume (LVESV), or left ventricular ejection fraction (LVEF), which were relatively preserved in both groups (Table 3).

We next examined CMR measurements of ventricular remodeling between baseline and follow-up scans within each individual participant (Table 3). There was an increase in LVEDV, decrease in LVESV, increase in LVEF, and a reduction in infarct size in both SMuRFless and SMuRF groups (*p* < 0.001 for all). The increase in LVEDV tended to be greater in SMuRF-less participants (*p* = 0.06). However, this was not significant when indexed for body surface area. The proportion of patients reaching ≥12% increase in LVEDV was equivalent in both groups. All other CMR ventricular remodeling measurements were similar between patients with or without SMuRFs (Table 3).

### Clinical outcomes

**Figure 3:**
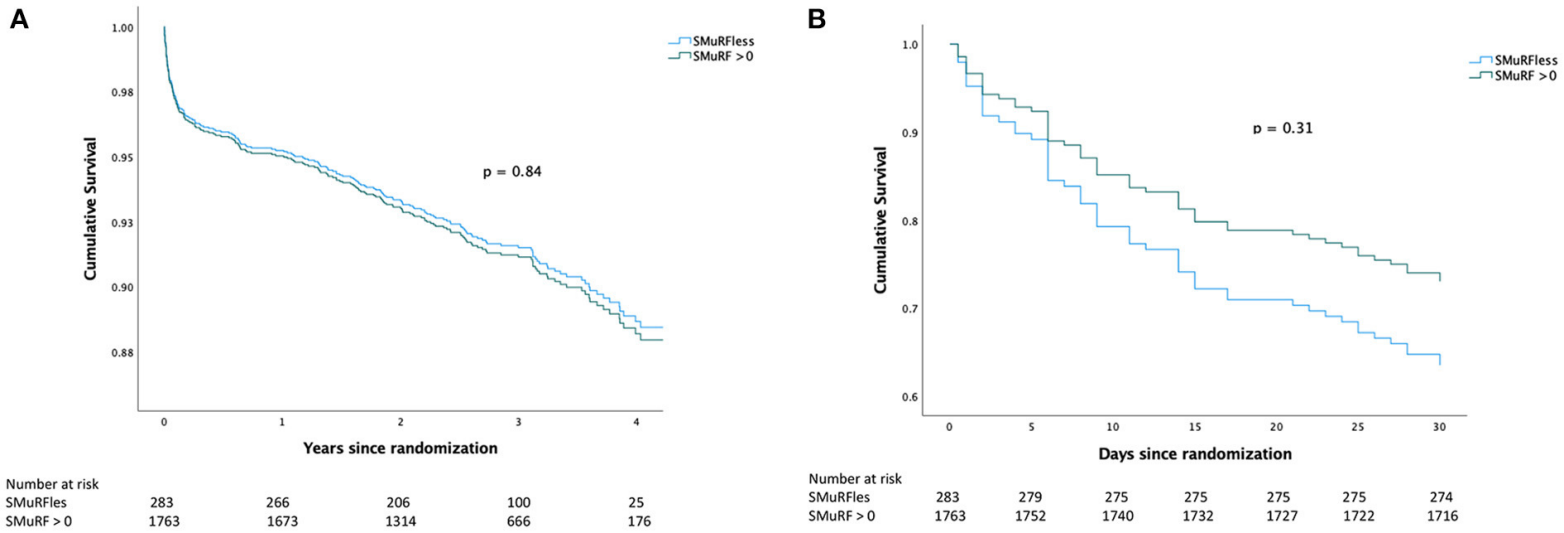
Kaplan-Meier curves. **(A)** Shows the Kaplan–Meier curve for the long-term composite endpoint of all-cause mortality and hospitalization for heart failure. **(B)** Shows the Kaplan–Meier curve for the composite endpoint of all-cause mortality and hospitalization for heart failure at 30 days.

The clinical outcomes at 30 days are presented in Table 6. Kaplan–Meier survival curves illustrating the 30-day risk and long-term risk of all-cause mortality and hospitalization for heart failure in patients with SMuRF vs. SMuRFless is shown in Figure 3. All the rates of all-cause mortality (3 vs. 2%), non-fatal reinfarction (0 vs. 1%), hospital admission for heart failure (1 vs. 1%), and the combined all-cause mortality/hospital admission for heart failure rate (3 vs. 3%) were low and similar between the two groups (SMuRFless vs. SMuRFs). Patients had a long-term clinical follow-up of a median of 3.1 years (IQR: 2.4–3.8) and no patients were lost to follow-up. Event rates continued to be low and there were no differences in any of the clinical outcomes at long-term follow up, when comparing SMuRFless patients to patients with >0 SMuRFs (Table 6).

**Table 6:** Clinical outcomes, total DANAMI-3 population.

	**SMuRFless** ***n* = 283 (14%)**	**SMuRF > 0** ***n* = 1,763 (86%)**	**Hazard** **ratio (95% CI)**	***p*-value**
30-days follow-up				
All-cause mortality, *n* (%)	8 (3)	31 (2)	1.7 (0.8–3.6)	0.20
Non-fatal reinfarction	0 (0)	26 (1)	0.04 (0.0–1.6)	0.30
Hospital admission for heart failure	2 (1)	20 (1)	1.1 (0.2–4.5)	0.94
All-cause mortality or hospital admission for heart failure, *n* (%)	9 (3)	47 (3)	1.4 (0.7–2.9)	0.31
Long-term follow-up				
All-cause mortality, *n* (%)	22 (8)	122 (7)	1.2 (0.7–1.8)	0.54
Non-fatal reinfarction	9 (3)	104 (6)	0.5 (0.3–1.1)	0.08
Hospital admission for heart failure	7 (5)	72 (4)	0.6 (0.3–1.3)	0.23
All-cause mortality or hospital admission for heart failure, *n* (%)	26 (9)	173 (10)	0.96 (0.6–1.4)	0.84

Data are number of events (%).

## Discussion

In recent studies, we have reported excess early mortality in STEMI patients who have developed CAD in the absence of modifiable risk factors. Contrary to previous publications, we did not find any difference in 30-day clinical endpoints. However, the CMR imaging of the DANAMI-3 trials allowed us to examine potential differences in acute infarct size and myocardial remodeling after the first presentation of STEMI in patients with no SMuRFs vs. those with at least one SMuRF. Whilst we did not find any difference in 30-day clinical endpoints, myocardial remodeling, or MVO in the DANAMI-3 study cohort, we did observe an increased acute and final infarct size in SMuRFless patients. This appeared to be at least partially mediated by a higher proportion of culprit locations in the LAD-territory and pre-PCI TIMI 0-1 flow among SMURFLESS patients.

SMuRFless patients are often an invisible subgroup in randomized clinical trials and subsequently, evidence-based guidelines for STEMI patients. Indeed, of the 256 controlled trials referenced in the ESC (European Society of Cardiology) and AHA (American Heart Association) STEMI guidelines, the proportion of patients with diabetes mellitus, hypertension, hypercholesterolemia, and smoking is a common feature of the baseline characteristics in Table 1, but not a single study reported the proportion of patients without SMuRFs (24–26). It is important to note that this proportion cannot be derived from the number of patients with one or more of the four SMuRFs reported. In the current study of 1st presentation STEMI patients in the combined DANAMI 3 studies, we confirm that a substantial proportion (14%) were unknown SMuRFless according to our definition. However, the proportion was smaller than initially reported (27%) (4, 5). In regard to their non-modifiable risk factors that may have contributed to their event, SMuRFless patients were older, were more likely to be male, and had a higher rate of prior smoking, but were less likely to have a family history of ischemic heart disease than their STEMI counterparts with at least one SMuRF. It is, however, important to bear in mind that the definitions of SMuRFs were based on electronic medical records and patients were categorized as having hypertension, hypercholesterolemia, or diabetes if the diagnosis was present at the time of enrolment or if LDL ≥3.5 mmol/L upon admission. Serum glucose was not incorporated in the diagnosis of diabetes due to the influence of AMI on glucose levels. Importantly, there may be a risk of patients neglecting to report their actual smoking habits leading to a falsely high number of patients reporting being prior smokers as well as unknown hypertension. We have accounted for this in the multivariable analyses.

In the recent study of the SWEDEHEART Registry, 30-day mortality rates were ~50% higher in SMuRFless STEMI patients (11.3%) vs. those with at least one modifiable risk factor (7.9%; *p* < 0.0001) (9). In comparison, the mortality rate was much lower overall in the DANAMI series (2%), and not significantly different between the groups. This lower mortality rate may reflect a degree of selection bias, with severely unwell patients being unsuitable for randomization to clinical trials, as well as potential higher rates of secondary prevention medications that are recognized to occur in the setting of a rigorous clinical trial vs. a “real-world” registry. It may also relate to the lower than expected proportion of women in the DANAMI study population, given women have an excess early mortality post STEMI (9, 27). On the other hand, register-based data are more prone to bias as regards the existence of comorbidities and risk factors, which may be even more pronounced among the most critically ill who also carry the highest mortality rate. The presence of multi-vessel disease in the overall population was higher in the SMuRF group (41 vs. 34%, *p* = 0.02), however, despite this the incidence of clinical outcomes like all-cause mortality, non-fatal reinfarction, or hospitalization for heart failure at 30 days or long-term follow up was not higher in the SMuRF group. Hence, the presence of higher multivessel disease in the SMuRF group probably did not have an effect on outcomes. The main emphasis of our study was the CMR subgroup to assess CMR imaging features in the two groups. In the CMR subgroup, there was no difference in the presence of multi-vessel disease between SMuRFs and SMuRFless patients (39 vs. 42%, *p* = 0.56).

The detailed CMR imaging data available enabled us to explore any potential differences in myocardial infarction and remodeling characteristics as a potential explanation of the early mortality. It could be hypothesized that factors involved in heightened susceptibility to coronary artery disease against a background of minimal risk factors may be a reflection of increased inflammatory and pathophysiological signaling downstream of the risk factors. Such signaling may also impact the myocardial response to a myocardial infarction and ischemia-reperfusion injury. However, this hypothesis cannot be confirmed in the present study as SMuRF was not an independent predictor of infarct size and myocardial salvage after adjusting for LAD territory and TIMI 0-1 flow.

The most significant predictors of infarct size were a culprit LAD lesion and TIMI 0–1 flow pre-PCI. This is consistent with the established literature, with extensive data demonstrating that STEMI due to LAD as the culprit vessel is associated with larger infarct size, worse left ventricle function, and higher mortality when compared with STEMI due to non-LAD arteries (28, 29). Interestingly, the proportion of patients with culprit LAD and TIMI 0-1 flow pre-PCI was also significantly higher in SMuRFless patients similar to what we had observed in the SWEDEHEART Registry (9), and CONCORDANCE (5) (Cooperative National Registry of Acute Coronary Syndrome Care) registry in Australia. The observed higher proportion of LAD culprit lesions in SMuRFless STEMI patients vs. those with at least one risk factor in SWEDEHEART (9) (42 vs. 37%) and CONCORDANCE (5) (46 vs. 37%) was initially considered to be due to chance. However, whilst chance may still be an explanation for the observation due to the many statistical comparisons, the consistent signal in the DANAMI series of LAD territory culprits in the SMuRFless group (50 vs. 42%) raises the possibility of an underlying biological mechanism that warrants further investigation (30, 31).

Poor pre-procedural TIMI flow in STEMI patients is associated with larger infarct size, higher fatal arrhythmia events, higher in-hospital, and one-year mortality (23, 32, 33). Some of the known predictors of pre-procedural TIMI 3 flow are high systolic blood pressure and fast heart rate on admission, diabetes, longer delay to PCI, smoking, and extensive coronary disease (23). Other studies have demonstrated prothrombotic and inflammatory markers like neutrophil to lymphocyte ratio, platelet count and reactivity, mean platelet volume, and uric acid levels to be associated with patency of infarct-related arteries before PCI (23). Plaque composition may also contribute to poor pre-procedural flow in STEMI patients. An intravascular imaging study of 111 STEMI patients demonstrated that patients with pre-procedural TIMI 0-1 flow had greater lipid burden, larger vessel size, and larger plaque areas (34). Interestingly, in our multivariate analysis of predictors of TIMI 0-1 flow pre-PCI, SMuRFless status was one of the independent predictors. Whether SMuRFless patients truly have a higher predisposition for the involvement of LAD and if there are any biological mechanisms predisposing them to a higher incidence of poor flow (TIMI 0-1) pre-PCI is unknown. Other important differences between the groups may also contribute to these observations e.g., age, gender, former smokers, and baseline cardiovascular medications (aspirin, statins, ACE -inhibitors, beta-blockers, etc.) with cardioprotective abilities prescribed due to the risk factors. It is possible that being on these medications pre-STEMI may provide a cardioprotective effect in patients with standard modifiable risk factors. Unfortunately, information on pre-STEMI prescribed medications is not available.

CMR is the gold standard for measuring left ventricle volumes and ejection fraction due to its high reproducibility (35). The DANAMI series inclusion of serial CMR allowed us to examine potential associations with adverse remodeling post-STEMI. The average increase in LVEDV tended to be larger in the SMuRFless STEMI patients than in those with at least one risk factor. No differences were seen in the change of ESV or LVEF over the 3 months between the groups. A previous study showed that adverse left ventricle remodeling after STEMI with ≥12% increase in both LVEDV and LVESV at 6 months was associated with worse clinical outcomes at 5 years in terms of all-cause mortality and hospitalization for heart failure in comparison to their counterparts (35). The proportion of patients reaching this cut off was equivalent in both our groups (11% of SmuRFless vs. 13% of patients with at least one SMuRF) (Table 3). There was no difference in MVO between the two groups. At baseline, the infarct size was larger in SMuRFless patients even after adjusting for the area at risk. Infarct size, as a percent of LV mass, decreased equally over the 3 months in both groups by 1.5%.

Although three different treatment strategies were used in the three randomized controlled STEMI trials of the DANAMI-3 trial program, there was no difference in clinical outcomes in these trials due to the interventions. Therefore, including patients from three different STEMI trials should not have influenced the clinical outcomes in our study. Furthermore, there was no interaction between the infarct size and either of the treatment arms. The limitations of the study include the low mortality rate and the potential for selection bias of a clinical trial, which contributing to this vs. real-world registries. This must be weighed against the benefits of prospectively collected data in randomized trials, which are less susceptible to missing data than registries. Many comparisons were made leading to the risk of type 1 error. Finally, many patients were lost to CMR, and it is well known that patients suitable for CMR are at lower risk compared to the CMR drop-outs.

## Conclusion

SMuRFless patients had a larger infarct size and smaller MSI following first-presentation STEMI, but this association was mediated by a higher proportion of LAD-territory events and pre-PCI TIMI 0-1 flow. Despite of this observation, mortality was not significantly different. Whether the finding of LAD territory and flow reduction preponderance in SMuRFless STEMI patients is a chance finding or related to unknown differences in biology remains to be examined.

## Data availability statement

The original contributions presented in the study are included in the article/supplementary material, further inquiries can be directed to the corresponding author.

## Ethics statement

The studies involving human participants were reviewed and approved by the Third DANish Study of Optimal Acute Treatment of Patients with ST-Segment Elevation Myocardial Infarction (DANAMI-3) trial program (NCT 01960933, NCT01435408; ethics approved by the Ethical Committee of the capital region of Denmark: H-4-2010-076. The patients/participants provided their written informed consent to participate in this study.

## Author contributions

GF, JM, SG, RK, and SV conceived the study. JL and KE conceived and designed the analysis approach. GF, JM, JL, and KE led the interpretation of the data for the work and drafted the work. RK, SG, LN-C, KA, HK, DH, LK, NV, and TE revised the manuscript critically and contributed important intellectual content. GF and JL approved the final version of the manuscript for publication and agree to be accountable for all aspects of the work. All authors contributed to the article and approved the submitted version.

## Funding

GF receives funding from National Health and Medical Research Council (Australia) Practitioner Fellowship (GNT1135920) and Center for Research Excellence (GNT1196629), and New South Wales Office of Health and Medical Research (Senior Investigator grant).

## Conflict of interest

Author LK reports Speakers honorarium from Novo, Novartis, AstraZeneca and Bohringer, unrelated to this manuscript. TE reports personal fees from Abbott, Bayer, and AstraZeneca outside the submitted work. GF reports personal consulting fees from CSL and grants from Abbott Diagnostic outside the submitted work. The remaining authors declare that the research was conducted in the absence of any commercial or financial relationships that could be construed as a potential conflict of interest.

## Publisher's note

All claims expressed in this article are solely those of the authors and do not necessarily represent those of their affiliated organizations, or those of the publisher, the editors and the reviewers. Any product that may be evaluated in this article, or claim that may be made by its manufacturer, is not guaranteed or endorsed by the publisher.

## Figures and Tables

**Table A1:** Randomizations, total DANAMI-3, and CMR sub-population.

**Total DANAMI-3 population**	**SMuRFless** ***n* = 283 (14%)**	**SMuRF > 0** ***n* = 1763 (86%)**	***p*-value**
**Primary randomization**			
Randomized to deferred stenting, *n* (%). (DEFER)	52 (18)	508 (29)	<0.001
Randomized to postconditioning, *n* (%). (iPOST)	104 (37)	472 (27)	<0.001
Randomized to conventional treatment, *n* (%)	127 (45)	783 (44)	0.88
**Secondary randomization**			
Randomized to full-revascularization, *n* (% within secondary randomization). (PRIMULTI)	26 (52)	267 (51)	0.85
**CMR sub-population**	**SmuRFless** ***n*** **= 61 (8%)**	**SMuRF > 0** ***n*** **= 665 (92%)**	***p*** **-value**
**Primary randomization**			
Randomized to deferred stenting, *n* (% within primary randomization). (DEFER)	17 (28)	222 (33)	0.38
Randomized to postconditioning, *n* (% within primary randomization). (iPOST)	19 (31)	160 (24)	0.22
Randomized to conventional treatment, *n* (%)	25 (41)	247 (42)	0.58
**Secondary randomization**			
Randomized to full-revascularization. (PRIMULTI)	10 (48)	136 (52)	0.22

**Table A2:** Medical therapy at discharge, total DANAMI-3 and CMR sub-population.

**Total DANAMI-3 population**	**SMuRFless** ***n*** **= 283 (14%)**	**SMuRF > 0** ***n*** **= 1,763 (86%)**	***p*** **-value**
Antiplatelet therapy, *n* (%)			
Aspirin	273 (96)	1,732 (98)	0.04
Clopidogrel bisulfate	74 (26)	266 (15)	<0.001
Prasugrel hydrochloride	70 (25)	946 (54)	<0.001
Ticagrelor	130 (46)	530 (30)	<0.001
Statin, *n* (%)	271 (96)	1,728 (98)	0.01
β-Blocker, *n* (%)	245 (87)	1,586 (90)	0.08
ACE/ARB, *n* (%)	125 (44)	799 (45)	0.71
**CMR sub-population**	**SmuRFless** ***n*** **= 61 (8%)**	**SMuRF > 0** ***n*** **= 665 (92%)**	**p-value**
Antiplatelet therapy, *n* (%)			
Aspirin	61 (100)	653 (98)	0.29
Clopidogrel bisulfate	3 (5)	40 (6)	0.73
Prasugrel hydrochloride	43 (71)	520 (78)	0.17
Ticagrelor	14 (23)	103 (13)	0.13
Statin, *n* (%)	61 (100)	661 (99)	1.0
β-Blocker, *n* (%)	58 (95)	613 (92)	0.41
ACE/ARB, *n* (%)	23 (38)	277 (42)	0.54
